# Investigating the Relationships between Basic Tastes Sensitivities, Fattiness Sensitivity, and Food Liking in 11-Year-Old Children

**DOI:** 10.3390/foods9091315

**Published:** 2020-09-18

**Authors:** Ervina Ervina, Ingunn Berget, Valérie L. Almli

**Affiliations:** 1 Nofima, Norwegian Institute of Food, Fisheries and Aquaculture Research, 1433 Ås, Norway; ingunn.berget@nofima.no (I.B.); valerie.lengard.almli@nofima.no (V.L.A.); 2 Department of Chemistry, Biotechnology and Food Science (KBM), The Norwegian University of Life Sciences, 1433 Ås, Norway

**Keywords:** taste sensitivity, basic tastes, fattiness, food liking, preadolescent, caffeine, quinine, gamification

## Abstract

This study investigates the relationships between basic tastes and fattiness sensitivity and food liking in 11-year-old children. The basic taste sensitivity of 106 children was measured using different methods, namely detection (DT) and recognition (RT) thresholds, and taste responsiveness. Caffeine and quinine (bitter), sucrose (sweet), citric acid (sour), sodium chloride (salty), and monosodium glutamate (umami) were investigated for DT and RT at five concentrations in water solutions. In addition, taste responsiveness and liking were collected for the high-intensity concentrations. PROP (6-n-propylthiouracil) responsiveness was tested on paper strips. Fattiness sensitivity was measured by a paired comparison method using milk samples with varying fat content. Liking for 30 food items was recorded using a food-list questionnaire. The test was completed in a gamified “taste detective” approach. The results show that DT correlates with RT for all tastes while responsiveness to PROP correlates with overall taste responsiveness. Caffeine and quinine differ in bitterness responsiveness and liking. Girls have significantly lower DTs than boys for bitterness and sweetness. Food liking is driven by taste and fattiness properties, while fatty food liking is significantly influenced by fattiness sensitivity. These results contribute to a better holistic understanding of taste and fattiness sensitivity in connection to food liking in preadolescents.

## 1. Introduction

Taste is of primary influence on food selection particularly in children [1,2,3]. It is one of the key factors determining food palatability and liking, which contribute to food intake [4]. Taste is also reported to affect food choices in children aged 12–13 and significantly determines food acceptance in 7–11-year-old children [5,6].

Children are individually different in perceiving tastes [7]. Taste sensitivity is defined as the individual ability in responding to taste stimuli [8] which could be measured by different methods such as detection threshold (DT), recognition threshold (RT), taste responsiveness, and fungiform papillae (FP) density [9]. In addition, sensitivity to PROP (6-n-propylthiouracil) has been associated with other taste responses, which makes this test commonly used in taste sensitivity studies [10]. The DT focuses on low concentrations of taste stimuli and is obtained at the point where the concentration of the taste stimulus can be discriminated from water [9]. As the concentration is further increased, RT is obtained as the point where the taste is perceived and identified [11]. Subjects can be separated into taste-sensitive and nonsensitive groups according to their DT or RT as previously proposed in adults [12] or in children aged 7–14 years [7]. Taste responsiveness is the subject’s rating of perceived intensity to taste stimuli above the threshold level, also known as the suprathreshold intensity [13]. The 6-n-propylthiouracil, also known as PROP, is a chemical compound commonly used to measure subjects’ responsiveness to bitterness [14]. However, responsiveness to PROP may not reflect responsiveness to all bitter compounds; this measure is specifically related to the genetic variants of taste receptor TAS2R38 for bitter perception [10,15]. Indeed, PROP is chemically similar to phenylthiocarbamide (PTC) and sensitivity to these compounds was reported to be associated with the TAS2R38 receptor [16,17,18,19]. Sensitivity testing to PROP can be conducted using water solutions [20,21] or impregnated paper [22,23]. People can be classified into supertasters, medium-tasters, and nontasters according to their responsiveness to PROP, i.e., their PROP phenotype. This classification is based on intensity ratings perception of the stimulus on a Labeled Magnitude Scale (LMS), and the application of established cut-off points that define the taster categories [23]. Subjects categorized as tasters perceive a higher bitter sensation of PROP compared to a nontaster [14,21]. The quantification of fungiform papillae (FP) has also been used to infer taste sensitivity. However, there is a concern related to using this method, as recent studies involving large population samples concluded that FP density does not directly correlate to taste sensitivity [24,25,26].

There are five basic tastes—sweet, sour, salty, bitter, and umami—that, respectively, relate to different receptors and mechanisms of responses [27]. In addition, the taste of fat (also called oleogustus) has been suggested as the sixth taste modality [28], with a gustatory pathway devoted to the perception of lipids [29]. The association between the different basic tastes and their taste receptors has been widely investigated, with the results suggesting that genetic variants may contribute to individual taste sensitivity [15,30]. Differences in taste sensitivity contribute to a variety of eating practices and food choices [31]. Taste sensitivity is shown to influence the willingness of 9–11-year-old children to consume fruits and vegetables [32] and significantly affects their acceptance of new foods [33]. Moreover, sensitivity to PROP is reported to affect the acceptance of sweet and fatty foods [34], and children aged 4–5 years with lower bitter taste sensitivity are reported to have a higher vegetable acceptance [35].

Regarding sweet taste, previous research has reported that sweetness sensitivity correlates with an increase in the liking of sweet food. This was investigated using sweetened apple juice in 6–9-year-old children [36]. The sweet sensitivity of 4–6-year-old children has also positively been correlated with their preference for sweetened beverages [37]. On the opposite end, a bitter taste has been associated with food rejection in children [38,39]. Moreover, 4–5-year-old children who were sensitive to PROP have been shown to have a lower acceptance for broccoli and cheese compared to nonsensitive children [21]. In a review on children’s perception of saltiness, Liem [40] concluded that saltiness plays an important role in children’s liking for a selection of salty foods, but that saltiness sensitivity does not influence children’s real consumption of salty food products. To our current knowledge, there are few studies about sourness sensitivity in children. Unlike the other basic tastes that consistently have been reported to influence food palatability, the literature indicates that sour taste does not significantly affect liking and preference in nine-year-old children, even after repeated exposure of this taste [41]. Moreover, a recent taste sensitivity study involving large samples of children aged 6–9 in Europe did not include sourness in the evaluations [42]. Further, umami has been reported to enhance palatability and acceptance of foods [43]. Sensitivity to umami has been reported to be significantly different in 13–16-year-old children according to their weight [44]. The study of the recognition threshold for this taste requires a training session [45] due to unfamiliarity and confusion between umami and saltiness [45,46]. In addition to the basic tastes, fattiness sensitivity has been highlighted to affect the liking and consumption of fatty foods [47,48]. However, the correlation between fat sensitivity and food liking in children seems to be inconsistent [49], particularly when weight status is involved. Previous investigations of fat sensitivity in children often used dairy samples such as milk, cheese, or pudding varying in fat content [50,51].

Although the matter of taste sensitivity and food liking has widely been investigated over the decades, it is still uncertain how different taste sensitivity measures relate to each other and to food liking. Moreover, different methods for measuring taste sensitivity may lead to different results, preventing easy results comparison between studies. A review by Cox et al. [49] suggested the need to measure the relationship between sensory sensitivity, fattiness, and liking. Earlier research results on the relationship between taste sensitivity and food liking are inconsistent [49] and studies involving preadolescent subjects are still limited [52]. The objective of this study is to investigate the relationships of basic tastes and fattiness sensitivity with food liking in 11-year-old children. By understanding the role of basic tastes and fattiness sensitivity in food liking, we may provide insights on how to encourage preadolescents to choose healthier food options, since this group has been reported to have selective eating [53]. To our knowledge, this is the first study on taste sensitivity investigating the relationship between five basic tastes as well as fattiness and food liking conducted in preadolescents. Moreover, different methods were applied including DT, RT, taste responsiveness, and PROP responsiveness testing to measure taste sensitivity. In addition, water solutions of both caffeine and quinine were utilized in this study to characterize sensitivity to bitterness, since subjects may have different sensitivity thresholds for different bitter compounds [54,55].

## 2. Materials and Methods

### 2.1. Participants

A total of 118, sixth-grade children were invited from two primary schools in Ski county, Norway. Both the children and their parents were provided with short information regarding the study activities in the form of a flyer. Signed written consent from the children and their parents was required to participate in the study. In addition, the children’s verbal consent was enquired at the beginning of the test. A total of 107 children returned the consent form and participated in the sensory testing. One of the subjects did not finish the test, resulting in 106 children involved in the data analyses. The schools received rewards for the benefit of the children for their participation, however, each child’s participation was voluntary. The ethical approval of this study has been granted by The Norwegian Center for Research Data (NSD) No. 747124 and refers to the Declaration of Helsinki, while data protection has followed the General Data Protection Regulation (GDPR). 

### 2.2. Test Procedure: The Taste Detective Game

When conducting sensory testing with children, it is important to implement a test procedure that is appropriate for the children’s age and psychosocial and cognitive ability [56]. In addition, it needs to be fun and engaging for them [52]. Therefore, a gamification concept was inserted into the testing procedure and introduced to the children as a game called the “taste detective”. In this sensory game, a short story was narrated, and the children were asked to conduct different tasks as taste detectives. The first task aimed to measure the children’s responsiveness and liking to basic tastes, the second task measured children’s basic tastes DT and RT, the third task measured fat sensitivity, and the last task measured children’s responsiveness to PROP. All the measurements will be explained in the following sections. To evaluate the gamification concept, the children rated how fun and how difficult the sensory game was at the end of the test. This was recorded on a seven-point pictorial scale labeled with “not fun at all!” to “very fun!” and “very difficult!” to “very easy!”.

The children’s age, gender, and self-reported hunger levels were also recorded. The children’s hunger levels were measured using a seven-point pictorial scale anchored from “not hungry at all” to “very hungry”. This practice was applied because previous research has shown that hunger may influence taste sensitivity [57] and the test was conducted at different times (10:30–11:15 and 11:30–12:15 for School A, and 12:30–13:15 and 13:00–13:45 for School B). Testing was conducted across 6 sessions with around 20 children for each testing time.

Most of the instructions were arranged online and the children’s responses were recorded with the aid of tablets. At the beginning of the test, we explained the rules of the game (i.e., performing the test quietly, not talking to one another, rinsing the mouth with water in-between samples, etc.) and what each task involved. It took the children around 30–45 min to finish all the tasks. All the tests were conducted in the children’s respective schools and classrooms. There were four adults present during the testing time: one person explaining the game and rules, two research assistants helping with the samples for the children, and one teacher.

### 2.3. Samples

The subjects’ basic tastes sensitivities to sweet (saccharose), sour (citric acid), salty (sodium chloride), umami (monosodium glutamate), and bitter (caffeine and quinine) were evaluated based upon five concentration levels each (Table 1). All the taste compounds are food grade and were purchased from Merck Kga, Germany. The samples were prepared by dissolving the tastant in tap water in the sensory laboratory at Nofima (Ås, Norway) a maximum of two days before the evaluation. Around 10 mL of the sample solutions were served to the children at room temperature.

The concentration levels for sweet, salty, umami, and caffeine-bitter followed the study from Knof et al. [58]. These concentration levels had been used in a large population study in Europe to measure taste sensitivity in 6–9-year-old children [42]. The sour taste concentrations followed the study from Myhrer et al. [59] while the bitterness level of quinine was adapted from Vennerød et al. [60]. All the levels of the sample solutions were first pretested by colleagues at the sensory department at Nofima, adjusted, then piloted with 42 children aged 11–12-years. The results showed that the selected sample solutions covered a suitable concentration range for measuring both DT and RT, and matched one another in concentration level intensity across the basic tastes (results not reported here).

### 2.4. Taste Responsiveness and Taste Liking

The children’s taste responsiveness was measured at the beginning of the test and using the strongest level (i.e., Level 5, see Table 1) of each taste compound in 10 mL servings. This level was expected to be clearly perceived by the majority of the children. The children evaluated all the basic tastes including two bitter compounds of caffeine and quinine in a randomized balanced order. Their responses were recorded in an unstructured line scale labeled with “weak” and “strong” and was then scaled into 0–100 for data analysis purposes. For this task, the cups were labeled with the names of the basic tastes. The liking of basic tastes in water solution at the same concentration level was also recorded in a seven-point pictorial hedonic scale. The children were provided a short explanation on how to use the line scale (i.e., by placing a mark on the line according to the strength of their perception after tasting the sample) and the pictorial scale (i.e., by choosing a happy or grim face according to the degree of their liking). This first session also aimed to familiarize the children with the basic tastes’ names and sensations. This was aimed to reduce the children’s confusion between the basic tastes in the following recognition task [61], particularly for salty-umami and sour-bitter [13]. Such confusions have been reported to often occur in children aged 7–11 years [45].

### 2.5. Detection and Recognition Test

The children’s taste sensitivity was also measured using detection and recognition thresholds, DT and RT. In this evaluation, they were told to solve six taste mysteries presented with different symbols. One symbol represented one basic taste and consisted of a series of five cups labeled from 1 to 5, corresponding to the increasing concentration levels of the taste (Table 1). All samples were given to the children at the same time in 10 mL servings, in addition to an identified cup of plain water for reference. The children were instructed to perform the tasting in a staircase order for one series (i.e., from Cup 1 to Cup 5) and could repeat their tasting for each cup. They had to identify the taste of each cup and would record their answers by dragging each corresponding cup on their tablet screen into the right taste box. Seven taste box options were offered: “sweet”, “sour”, “salty”, “bitter”, “umami”, “water”, and “I don’t know”. Note that all seven answer options were available for each cup at any time during the whole test. For each specific concentration level, we assumed that children who answered “water” could not detect any taste (tastant under detection threshold). DT was obtained when the subject could start to differentiate the sample from water, while RT was obtained when the child correctly identified the taste. Last, we assumed that children who either answered “I don’t know” or wrongly identified the taste quality, could detect the tastant; this level was therefore recorded as their DT. On their tablets, the children could freely place each cup in any taste box according to their own perception, without any limitation regarding the number of cups that could be placed in each box. Moreover, the children were not told that each series of five cups all carried the same taste, so they could freely attribute different tastes to cups of a given series. Once a taste series of five cups was completely evaluated, a break was provided with a few items from the food liking questionnaire (see below). Then, the on-screen instructions indicated to the child which symbol they should categorize next. It was not possible for the child to reconsider cups from the previous symbols.

In this test, we informed the children that there were no right and wrong answers, as this depended on their own perception. This point was strongly reminded and inserted as one of the game rules. Moreover, the children had to compare the samples with water and to rinse their mouths between tasting. They could spit out the samples in spitting cups to avoid being bloated during the evaluation. The taste series were tested in a randomized balanced order across children.

### 2.6. PROP Responsiveness

PROP responsiveness was measured by a paper strip (Precision Laboratories, Inc., Northampton, United Kingdom). The use of this paper strip was adapted from a method by Pickering et al. and Oftedal and Tepper [23,62]. The children were asked to place the strip in their mouth and hold it for 30 s before rating the bitterness intensity using the LMS [63]. Prior to this task, the children were provided with the instructions on how to use the LMS by using examples of foods that have extreme and mild sensations such as syrups and mineral water, salted potato chips and a spoon of salt, a spoon of wasabi, etc. [64]. The children were classified based on their LMS rating into nontasters (if they rated the bitterness ≤ 13 mm on the LMS), medium-tasters (14–67 mm), and supertasters (>67 mm) [23]. The test was allocated at the end of the whole testing session to refrain supertasters from being demotivated for further participation. The children received water and fresh fruits (grapes) after this task to clear their mouth from any unpleasant lingering sensation.

### 2.7. Fattiness Sensitivity Test

A paired comparison method adapted from Alexy et al. [50] was used to measure fattiness sensitivity. Four milk samples were tested in pairs with 0.5% (low), 1% (medium), and 1.5% (high) fat content differences for each pair (Table 2). All the milk samples were purchased from a local supermarket. There was no modification to the fat content for each milk sample except for the 2% fat milk, which was obtained from mixing the 3.5% and 0.5% samples in a 1:1 ratio. The milk pairs were presented in disposable cups and labeled with a geometric symbol, followed by a unique three-digit random number for each milk sample. The children’s task was to identify the fattiest milk sample in each pair, in addition to the sample they liked the most. To explain the fatty taste, the children cited examples of fatty foods (i.e., cream, butter, etc.) prior to the evaluation. All the pairs were served at the same time. Both the pairs and the milk samples within pairs were tested by the children in a randomized order. The children were told to rinse their mouth with water between testing the milk samples. Those who reported having a milk allergy and/or lactose intolerance or who declined to taste the milk samples were excluded from the milk evaluation (19 excluded, leading to *n* = 87 subjects who completed the task). 

### 2.8. Food Liking Questionnaire

The children completed a food liking questionnaire which consisted of 30 different food items representing five different basic tastes, and fattiness (the list of the food items is presented in Supplementary Material Table S1). The selected food items and their basic tastes and fattiness profiles were based on a study by Martin et al. [65], and were relevant within the Norwegian diet as they were listed in the Norwegian dietary survey [66]. The children were asked about the familiarity of five random food items by either stating “I have tasted it” or “I have never tasted it”. If they had tasted the item before, they then scored their liking using a seven-point pictorial hedonic scale. These practices were conducted six times between the basic taste sensitivity measurements, aimed to provide a short break from the tasting task as well as reducing boredom to cover the list of 30 food items. The food items were evaluated in a randomized balanced order.

### 2.9. Data Analysis

Multiple Factor Analysis (MFA) was applied to explore the relationship between taste sensitivities measured by different methods. The liking of the basic tastes in water solutions (Level 5 concentration) was included in the MFA as supplementary variables.

The overall DT, RT, and taste responsiveness scores were computed by averaging DT levels, RT levels, and taste responsiveness scores, respectively, across the six compounds tested in water solutions. This was aimed to observe the relationship between each measurement of taste sensitivity. Pearson correlations were computed between the different sensitivity measurements and between taste compounds. The different taste sensitivities (DT, RT, and taste responsiveness), as well as the liking of basic tastes in water solution, were modeled using linear mixed models. This analysis was aimed to explore the effect of taste quality, hunger level, and gender (fixed effects) on taste sensitivity or liking of basic taste in the water solution sample. In these models, a child nested within gender was included as a random effect. In addition, PROP responsiveness was included as a continuous variable. The children’s hunger levels across schools and testing times were also compared and analyzed using a Student’s *t*-test.

Linear mixed models were also applied to test the effect of taste sensitivity on food liking. Taste sensitivity (as measured by DT or taste responsiveness), taste quality, gender, and hunger level were included as fixed factors, whereas child nested within gender was included as a random effect. In addition, PROP was added as a continuous variable. Note that RT was not investigated in such a model as it was not shown to be influenced by any of the explanatory variables from the previous mixed model analysis. Further, Principal component analysis (PCA) was applied to map the children’s food liking with the children’s liking scores as columns and food items as rows. The food liking scores were double-centered prior to the analysis as this enables us to better observe individual differences [67]. The taste profiles of the foods were coded as binary variables for each of the basic tastes and fattiness (+1 if present, 0 if not present) and included as supplementary variables. The children were grouped into three liking groups based on PC1 and PC2 loadings, and a two-way ANOVA was then conducted to analyze the group effect and gender effect on taste sensitivity measured by DT for each taste.

Based on their response in identifying the correct milk pair, the children were categorized into sensitive and nonsensitive groups with respect to fattiness. The children who correctly answered the pairs from low- to high-fat levels in a staircase order (as seen in Table 2) were allocated to the fat-sensitive group (this includes those who correctly identified all the pairs; those who correctly identified both medium and high pairs; and those who correctly identified the high-fat pair only). The remaining children (those who answered all pairs incorrectly, or those who answered other than the above-mentioned pattern) were categorized as the non-fat-sensitive group. This practice was carefully applied to eliminate the chance of guessing and inconsistent answers from the children, as our data showed an inconsistency from several children who correctly classified the low-fat pair (0.5%) but were not able to identify the high-fat pair (1.5%). The effect of fattiness sensitivity on the liking of fatty foods between the groups was analyzed using Analysis of Covariance (ANCOVA), with PROP involved as a continuous explanatory variable.

Pairwise comparisons were conducted using Tukey’s post hoc test with a significance level set to *α* = 0.05. All statistical analyses were computed using XLSTAT Sensory version 2020.1.2 (Addinsoft, France).

## 3. Results

### 3.1. Subject Characteristics and the Taste Detective Game Approach

Forty-six percent of the participants were boys while 54% were girls. Ninety-four percent of the children were 11-year-old (mean age = 10.9 years). There was no significant difference regarding the children’s hunger level between different schools and testing times. Moreover, the hunger level did not contribute to a significant effect in any of the models and was therefore excluded from further results. The taste detective game was rated as a fun activity by 84% of the children (Table 3) and 63% of the children rated the game as easy to conduct.

### 3.2. Children’s Basic Tastes Sensitivity and Liking in Water Solutions

**Figure 1:**
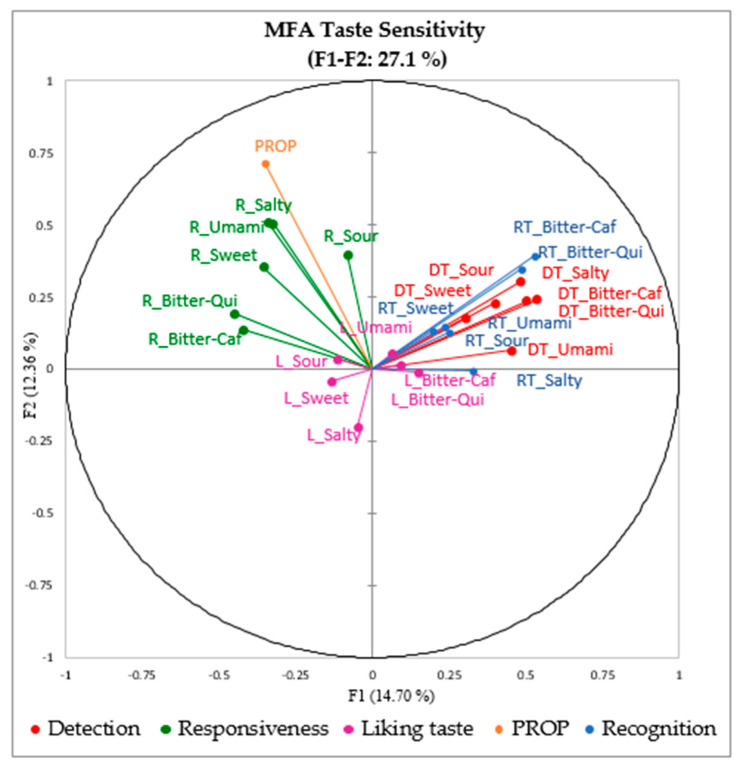
MFA of taste sensitivity and liking measures, included as supplementary variables (R = responsiveness, DT = detection threshold, RT = recognition threshold, L = liking of taste solutions).

The classification of children’s taste sensitivity based on the PROP phenotype resulted in 13% nontasters, 51% medium-tasters, and 36% supertasters. Furthermore, the first two factors in MFA weighed for 27.1% of the variability (Figure 1). The DT showed to be strongly correlated with RT while the taste responsiveness had a high correlation with PROP responsiveness in the MFA map, indicating that DT and RT seemed to measure a different dimension of sensory perception from taste responsiveness and PROP. The liking of the Level 5 basic taste solutions (see Table 1) did not correlate well with any of the sensitivity measures, indicating that this affective response to taste is only partially dependent on objective detection, recognition, and responsiveness measures. The sweet and sour tastes were recorded as the most liked while umami and salty were the most disliked from the water solution samples (Table 4). Gender had a significant effect on the liking of basic taste in the water solutions, indicating a higher liking for sweetness (*p* = 0.004) and bitterness of quinine (*p* = 0.07) for boys compared to girls.

Based on Pearson correlation coefficients (*r*), all the taste sensitivity measurements were significantly correlated to one another, except for PROP responsiveness and RT. There was a significant positive correlation found between overall DT and RT scores (*r* = 0.30, *p* < 0.001), and between overall taste responsiveness and PROP (*r* = 0.13, *p* = 0.001), with each of the basic tastes individually showing similar results. Taste responsiveness to salty and to umami showed a significant positive correlation with PROP responsiveness. Moreover, overall DT and RT were negatively correlated with taste responsiveness (*r* = −0.14, *p* = 0.001 for DT; *r* = −0.11, *p* = 0.006 for RT). We may however note that although significant, all the correlations found were rather weak.

Table 4 summarizes the children’s taste sensitivity and liking for each basic taste, as measured by the different methods. Regarding taste responsiveness, the umami taste triggered the most intense sensation (mean: 53.8 mm) followed by the sweet taste (mean: 44.6 mm). Quinine showed the highest standard deviation (mean: 36.9 mm, SD = 31.8), indicating that this compound was most subject to individual variations. Further, the salty taste showed to have the lowest detection threshold level (DT_salty_ mean: 1.5, equivalent to 0.32 ± 0.20 g/L sodium chloride) followed by the sweet taste (DT_sweet_: 1.6, i.e., 4.78 ± 2.09 g/L saccharose). Moreover, bitterness had the highest DT levels, with mean detections over Level 2 (DT_caffeine_: 0.12 ± 0.07 g/L; DT_quinine_: 0.002 ± 0.001 g/L). Concerning the RT level, the sweet taste showed to be the lowest (RT_sweet_ mean: 3.2, i.e., 9.81 g/L saccharose) while the bitter tastes from caffeine and quinine were the highest. Sweetness and bitterness from caffeine seemed to be the easiest tastes to name correctly once perceived, with the lowest mean differences RT-DT of 1.6 levels. On the contrary, saltiness and umami seemed to be the hardest tastes to name correctly with a mean RT-DT of 1.9 levels. In terms of concentration of the taste compounds, on average sweet was detected at a concentration of 4.78 g/L while quinine was already detected at 0.002 g/L (Table 4). 

**Figure 2:**
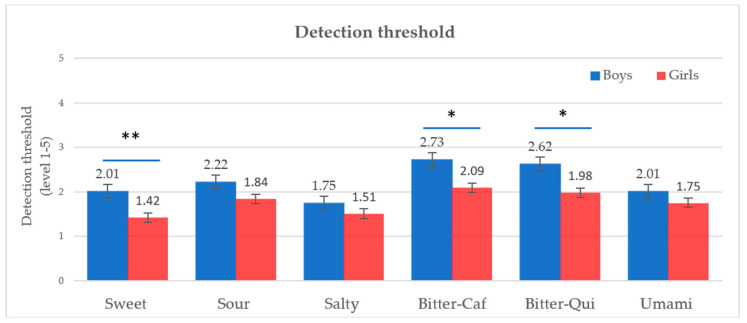
Detection threshold according to gender (* *p* < 0.1, ** *p* < 0.05, *t*-test).

The linear mixed model of taste sensitivity showed that taste quality was the most significant factor influencing taste responsiveness and DT (*p* < 0.001), but not RT (*p* = 0.189). Moreover, PROP responsiveness had a significant effect on taste responsiveness (*p* = 0.026), but this was not observed on the DT and RT. Gender appeared to marginally influence the DT (*p* = 0.08), showing a higher DT for boys than girls particularly for sweetness and bitterness of both caffeine and quinine. This indicates a lower sensitivity for the sweet and bitter tastes in boys compared to girls (Figure 2).

The sensitivities to bitterness from caffeine and quinine had significant moderate correlations for responsiveness (*r* = 0.47, *p* < 0.001), DT (*r* = 0.38, *p* < 0.001), and RT (*r* = 0.36, *p* < 001). On average, there were no differences observed for DT and RT levels between these two bitter compounds. However, the children’s responsiveness to bitterness was significantly higher for quinine than for caffeine (Table 4; *p* = 0.006). In addition, the liking of these two compounds was also perceived to be significantly different (*p* = 0.037). These results indicate different responses to bitterness from caffeine as compared to quinine. Finally, PROP responsiveness was not correlated with any measured bitterness sensitivity of caffeine and quinine, indicating a different bitterness sensitivity between PROP, caffeine, and quinine.

### 3.3. Children’s Stated Food Liking

**Figure 3:**
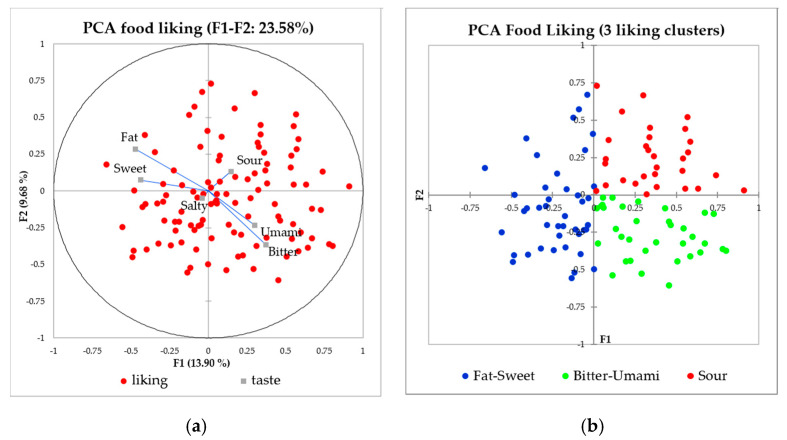
Loading from PC1–PC2 of the double-centered PCA on food liking (**a**) resulting in three taste-liking clusters of fat-sweet (*n* = 42), bitter-umami (*n* = 34), and sour (*n* = 30) likers (**b**).

The children’s reported liking of the listed food items was significantly higher for foods typical of sweet (mean liking 6.1 on a 1–7 scale) and fatty (mean = 5.9) characteristics, while foods characterized by the bitter (mean = 4.3) and umami (mean = 4.9) tastes were the least liked. The salty (mean = 5.6) and sour (mean = 5.5) foods were scored above bitter and umami foods. The children’s liking score was then analyzed using a double-centered PCA. The first two principal components accounted for 23.6% of the variability. Based on the PCA, the children were divided into three groups of fat-sweet (40%, *n* = 42), sour (28%, *n* = 30), and umami-bitter (32%, *n* = 34) liking (Figure 3). Salty was neither clearly presented in the first two nor in later principal components.

**Figure 4:**
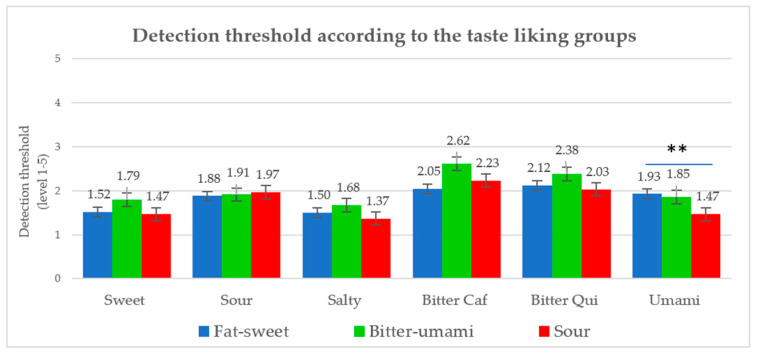
Mean detection threshold level for the three taste-liking groups (Caf = caffeine, Qui = quinine, ** *p* < 0.05 from ANOVA test).

The differences between the three liking clusters with respect to DT were analyzed using a two-way ANOVA. DT for the food liking clusters were significantly different for the umami taste only (Figure 4, *p* = 0.024), with the highest DT (least sensitivity) observed for the fat-sweet group and lowest for the sour group. Moreover, the bitter-umami group had higher DT for sweetness, saltiness, caffeine-bitter, and quinine-bitter compared to the other groups, but without reaching statistical significance (Figure 4). The linear mixed models showed no influence of DT and taste responsiveness on the children’s food liking, the only strong effect observed was from the different taste qualities (*p* < 0.001). Moreover, neither bitterness sensitivity from caffeine nor from quinine significantly influenced the liking of the bitter foods.

### 3.4. Fattiness Sensitivity and Liking of Fatty Foods

Results from the pairwise milk samples comparison test (see Table 2) show that 49% of the children correctly identified the fattiest sample in the low-fat milk pair and the medium-fat pair, and 56% in the high-fat pair (Table 5). The data show that the children who were best able to distinguish the milk with the highest fat content typically preferred low-fat milk. This was observed in all pairs of the low-fat (70% of the children preferred low-fat milk), medium-fat (60%), and high-fat (61%) milk. This indicates that more fat-sensitive children tend to prefer low-fat milk samples. Moreover, in all pairs, children who were not able to differentiate the milk samples analytically tended to prefer the high-fat milk sample, indicating that non-fat-sensitive children tend to prefer high-fat milk. Further, the clusters according to the fattiness sensitivity in milk resulted in 42.5% (*n* = 37) of the children being categorized into the fat-sensitive group and 57.5% (*n* = 50) in the nonsensitive group. The ANCOVA analysis showed that the nonsensitive group had a significantly higher liking (*p* = 0.04) to fatty foods (mean liking = 6.1 ± 0.5 SD) than the fat-sensitive group (mean liking = 5.8 ± 0.8 SD). We may also note that in this analysis no effect of PROP sensitivity on the liking of fatty food was revealed.

## 4. Discussion

The objective of this study was to investigate the relationships between basic tastes and fattiness sensitivity and food liking in 11-year-old children. A comprehensive approach was adopted including five basic tastes as well as fattiness, investigating bitterness through three bitter compounds, and utilizing four different methods to measure taste sensitivity in addition to fattiness. The different findings in our study are summarized in Table 6 and discussed below.

### 4.1. Basic Tastes Sensitivity in Children

#### 4.1.1. Relationships between Taste Sensitivities Measured by Different Methods

In our study, we found a negative correlation between taste responsiveness and the DT and RT, while PROP responsiveness showed to be positively correlated with overall taste responsiveness (Table 6). We also found that all measured taste sensitivities were significantly correlated, except for RT and PROP responsiveness. This was true for all taste qualities. These relationships between taste sensitivities are aligned with previous investigations on adults [9,68]. In particular, Dinnella and colleagues [24] reported a positive correlation between PROP and taste responsiveness in a large population sample of adults. In our study, however, these correlations seem to be weak, corroborating previous studies [11,68,69]. This demonstrated that except for DT and RT, taste sensitivity measurements are not strongly correlated with one another as each method captures somewhat different aspects of taste sensitivity [11]. Based on our results, taste responsiveness and DT were shown to better differentiate children’s taste sensitivity. It has been suggested by Fischer et al. [16] to measure directly perceived intensity for each taste, rather than using PROP responsiveness as a global indicator of taste responses.

Taste quality was the most significant factor influencing taste responsiveness and DT. However, this effect was not observed in RT. One explanation could be the number of concentration levels used. In our study, five levels were used to investigate both DT and RT. This is fewer than the eight levels used in ISO 3972 [70] for measuring basic taste recognition thresholds. However, the use of more taste levels could lead to a limitation as this practice may result in the children becoming fatigued [52]. Sensory testing with children has to be performed in the shortest possible time since they have a shorter attention span than adults [71].

#### 4.1.2. Bitterness Sensitivity to Caffeine, Quinine, and PROP 

Keller and Adise [72] classified a general adult population of approximately 25% nontasters, 50% medium-tasters, and 25% supertasters. However, this general classification is highly subject to factors such as age, gender, ethnicity, and health status [72,73]. In the present study, the classification of the children’s taste sensitivity based on PROP test strips led to 13% nontasters, 51% medium-tasters, and 36% supertasters (Table 6). This distribution is in accordance with a previous taste sensitivity study that reported the clusters of the taste phenotype consisting of 7% nontasters, 59% medium-tasters, and 34% supertasters in 13–17-year-old children [74]. Additionally, Mennella et al. [75] also concluded that there are age differences in PROP responsiveness, suggesting that when matched for the TAS2R38 genotype, children tend to be more sensitive than adults.

In our study, we found significant moderate correlations of bitterness sensitivity between caffeine and quinine regarding their DT, RT, and responsiveness, while no correlation was found between the bitterness sensitivity of caffeine or quinine with PROP responsiveness (Table 6). This demonstrates individual differences for bitterness as previously investigated in adults [54,55]. In these previous studies, the bitterness profiles of caffeine and quinine formed the same cluster, while the bitterness of PROP did not cluster with any other bitter compound [54]. Moreover, responsiveness to bitterness from different bitter compounds was reported to be different across adult subjects [55], as different compounds vary in their capacity to stimulate TAS2R bitter receptors [76]. Caffeine and quinine do not activate the TAS2R38 bitter taste receptor like PROP does [77,78], which indicates no genetic correlation between the bitterness perception of quinine or caffeine with PROP. This fact underlines the prevalence of individual differences for bitterness sensitivity to these compounds. Further, our results highlighted intraindividual differences in the responsiveness to and liking for caffeine as compared to quinine. Indeed, using the time-intensity method, Jane and Noble [79] compared caffeine and quinine and showed that caffeine had a longer bitter aftertaste and elicited a faster rate for maximum bitter perception than quinine, indicating different bitterness profiles between these two compounds. In summary, the different profiles, and mechanisms at play in the perception of caffeine, quinine, and PROP lead to both inter- and intraindividual differences and should be considered when selecting bitter compounds to represent bitterness in sensory studies. 

#### 4.1.3. Gender Effect on Taste Sensitivity

In our study, gender was shown to influence the children’s DT, indicating a lower taste sensitivity for boys than girls, with the most evident differences observed in sweetness and bitterness (Table 6). However, gender did not affect taste responsiveness. The differences in taste sensitivity between genders remain controversial [80]. Spence [81] reported no differences between men and women in perceiving taste. It was also suggested that differences between men and women to chemosensory stimuli were due to different cognitive evaluations rather than sensory sensitivity [82]. However, our result corroborates with a previous study by Joseph and colleagues that reported significant differences in taste sensitivity based on the detection threshold in 7–14-year-old male and female subjects [7]. In this study, gender differences were reported for sweetness with lower DTs in girls compared to boys. Previous studies involving a large adult population also highlighted that women were significantly more sensitive than men for sweet, sour, salty, and bitter taste stimuli [83,84].

### 4.2. Children’s Taste Sensitivity and Food Liking

Based on a list of 30 food items, the children showed a greater stated a liking for typically sweet and/or fatty foods than for foods characterized by other taste qualities (Table 6). This result corroborates a previous study by Dieuwerke et al. [85], which revealed that sweet and fatty tastes provide the strongest influence on food liking. Their study used tomato soups and custard samples, with varying degrees of sugar and fat. Moreover, a study involving more than 1800 children aged 6–9 years also reported that children significantly prefer sugar-sweetened apple juice and fat-enriched crackers [42]. 

In our study, typical bitter or umami food items were less liked than typical sweet, fatty, salty, and/or sour foods. Bitter taste triggers a rejection response in children [86] and a similar rejection was reported for umami taste in water solutions [87]. The umami taste alone (monosodium glutamate) is not palatable, therefore the children rated this taste as the most disliked in the water solution sample. However, the combination of umami with other tastes and flavors including saltiness and fattiness can create a pleasant savory perception [43]. The dislike for umami foods in our study might be due to the unfamiliarity of this taste since we record a lower familiarity for bitter and umami foods compared to other foods (Supplementary Material Table S1). Umami taste was previously categorized as an unfamiliar taste [46] and the unfamiliarity of this taste was reported to be even stronger in children aged 7–11 [45], which could explain the low acceptability of this taste.

There was no strong relationship between taste sensitivity and food liking in our study. One possible explanation is that the typical taste in food will generally lie above the detection threshold [13], while the not-so-typical, low-intensity tastes would solely be perceived by the most sensitive children. The only significant effect found was for umami sensitivity (DT) indicating that the children’s taste sensitivity differed the most for this taste compared to the other basic tastes. In line with this finding, previous research has reported that individual differences in taste sensitivity vary the most for umami taste [31], as this taste has been found to have various recognition and hedonic responses [46]. Moreover, evidence for genetic variation for this taste has been revealed [88] and umami sensitivity has been reported to be significantly different in preadolescents [44]. 

According to Puputti et al. [89], taste sensitivity influences food consumption but there is no effect between taste sensitivity and food liking, also corroborating Tepper’s study [10]. This association was investigated for the first time by Pangborn and Pecore [90] who aimed to understand the correlation between DT and hedonic response. The results showed that taste acuity stands in a different dimension from the hedonic response and these two measures may not directly explain one another. This indicates that taste sensitivity might not directly link to the food liking.

Indeed, food liking in children could not be influenced by taste sensitivity alone. Previous research has highlighted that other factors such as familiarity showed to significantly affect children’s liking as children will eat what they like, and like what they know [91,92]. Extrinsic factors such as the family’s socioeconomic and cultural background have been reported to also provide a significant effect on children’s food liking [93]. Moreover, in our study, taste sensitivity was measured using a water solution sample, but food liking was measured by a stated liking (without tasting) in a questionnaire. This may influence the liking for each food since children have their own internal scripts and experience regarding how these foods are cooked and served [94]. 

### 4.3. Fattiness Sensitivity and Food Liking

The nonsensitive group showed to have a higher liking of fatty foods compared to the fat-sensitive group. Moreover, we also found that children who correctly identified the fatty samples in milk pairs, preferred the low-fat milk samples while the children who incorrectly identified the milk pairs preferred the high-fat milk samples (Table 6). These findings show that fattiness sensitivity influences milk preferences and significantly affects selected fatty food liking in preadolescents. The results are in line with a previous study by Bolhuis and colleagues who reported a higher acceptance for low-fat tomato soups in the fat-sensitive group compared to the nonsensitive group [95]. Similarly, Liang et al [96] underlined a higher liking for fatty foods from subjects who were not sensitive to fattiness. Furthermore, according to our results, the liking of fatty foods was not related to PROP responsiveness. This corroborates several previous studies that did not find a strong correlation between fattiness liking and PROP responsiveness [97,98,99].

### 4.4. Methodological Approach and Limitations of the Study

To our knowledge, this is the first study investigating taste sensitivity in 11-year-old children in depth, with the combination of four approaches to sensitivity measurement (detection threshold, recognition threshold, taste responsiveness, and responsiveness to PROP), the inclusion of three bitter compounds (caffeine, quinine, and PROP), and the investigation of all five basic tastes as well as fattiness in the same study. Moreover, we study relationships between sensitivity measures and taste liking in water solutions as well as food liking from a questionnaire.

The use of gamification in sensory testing has been reported to improve the participation rate of children and make them interested to join [52]. In our study, the children were excited and engaged with the sensory testing being performed as a game. This could be seen by the low dropout rate of the children as only one child was not able to finish the test despite the high number of samples that had to be evaluated (i.e., 36 water solutions and 6 milk samples in total). Moreover, the sensory testing activity was rated as fun and easy to follow by the children. The aid of technology such as the online test setup and using tablets to record the children’s responses simplified the test instructions and data collection [71].

Some limitations of our approach may be noted. Our study may suffer from cross-modal correspondence effects from the symbols that we have used to mark the samples. Symbols of natural elements (a cloud, a moon, a flower, a sun, a star, and a leaf) were used to symbolize sweet, sour, salty, caffeine-bitter, quinine-bitter, and umami, respectively. The cross-modal correspondence between visual and taste stimuli has been reported to influence children’s perception [100]. This effect, however, was not investigated in our study. Furthermore, taste sensitivity results are directly dependent on the concentration levels of the chosen taste compounds. We strained to develop five comparable concentration ranges across tastants based on previous literature and extensive pilot testing, yet other results may be obtained from different concentrations and different taste stimuli choices. In addition, PROP responsiveness was investigated using a paper strip while the other taste compounds were evaluated in water solution samples, and the fattiness sensitivity was measured using a food sample (milk). All these nuances may influence the children’s responses due to different matrices and appearances of the taste stimuli. Finally, the fat sensitivity test was conducted after the taste sensitivity evaluation. This may result in the children becoming fatigued, possibly leading to a higher number of children categorized in the nonsensitive fat group. In addition, few pairs of milk samples were presented, and no repetition was conducted for fat sensitivity measurement, leading to a somewhat unsecure classification in fat-sensitive and nonsensitive groups, which we also consider as a limitation of our study.

## 5. Conclusions

This study aimed to investigate the relationship between basic tastes and fattiness sensitivity and food liking in preadolescents. The taste sensitivities measured with different methods showed to be significantly correlated to one another except between PROP responsiveness and RT. According to our results, DT and taste responsiveness were able to better differentiate children’s taste sensitivity compared to RT and PROP. Boys showed to have a lower taste sensitivity than girls according to their detection threshold for sweet and bitter tastes. Interestingly, the two bitter compounds of caffeine and quinine investigated in this study showed to be only moderately correlated in sensitivity, and perceived differently in terms of responsiveness and liking. This exhibits individual differences in bitter compounds sensitivity in preadolescents, and highlights the need to carefully consider different bitter compounds for future studies in taste sensitivity. Moreover, the fattiness sensitivity showed to significantly influence the liking of fatty foods in 11-year-old children. Our results showed no significant influence of taste sensitivity on the children’s food liking for the selected food items. The children’s food liking was observed to be strongly driven by different taste qualities and fattiness. These results contribute to a better holistic understanding of taste and fattiness sensitivity in connection to food liking in preadolescents.

Future research may investigate the relationship between taste sensitivity and food preferences using real or model food samples. The use of real/model food is expected to improve the relevancy of studying taste perceptions instead of measuring stated liking (without tasting) and water solutions. In addition, other factors that might influence taste sensitivity in preadolescence such as food exposure also need to be considered.

## Figures and Tables

**Table 1:** Basic taste concentration levels.

Taste	Taste Compound	Level 1 (g/L)	Level 2 (g/L)	Level 3 (g/L)	Level 4 (g/L)	Level 5 (g/L)
Sweet	Saccharose	3.0	6.0	9.0	12.0	16.0
Sour	Citric acid	0.05	0.1	0.16	0.2	0.25
Salty	Sodium chloride	0.2	0.4	0.8	1.2	1.6
Umami	Monosodium glutamate	0.1	0.3	0.6	1.2	1.5
Bitter	Caffeine	0.05	0.1	0.15	0.2	0.27
Bitter	Quinine	0.0014	0.0017	0.0023	0.0038	0.006

**Table 2:** Milk samples for the fat sensitivity test.

Pair	Fat Content Differences	Samples
Low	0.5%	0.5% fat milk
		1.0% fat milk
Medium	1.0%	1.0% fat milk
		2.0% fat milk
High	1.5%	2.0% fat milk
		3.5% fat milk

**Table 3:** The participants’ characteristics and evaluation of the game.

Variables	Participants (*n* = 106)
Gender	Boy 46% (*n* = 49)
	Girl 54% (*n* = 57)
Age	10-year-old 5% (*n* = 5)
	11-year-old 94% (*n* = 100)
	12-year-old 1% (*n* = 1)
PROP (6-n-propylthiouracil) status	Nontaster 13% (*n* = 13)
	Medium-taster 51% (*n* = 55)
	Super-taster 36% (*n* = 39)
Hunger level (scale 1–7) *	School A (*n* = 61) 3.5 ± 1.4 SD
	School B (*n* = 45) 3.9 ± 1.9 SD
Enjoyment of the game	Not fun 6% (*n* = 6)
	So so 10% (*n* = 11)
	Fun 84% (*n* = 89)
Difficulty of the game	Difficult 15% (*n* = 16)
	So so 22% (*n* = 23)
	Easy 63% (*n* = 67)

* No significant difference between schools and testing times (*p* > 0.05).

**Table 4:** Children’s taste sensitivity (responsiveness, DT, and RT) and liking of basic taste solutions.

Taste	Responsiveness (mm; Mean ± SD)	Detection (Mean ± SD)		Recognition (Mean ± SD)		Liking ^3^ (Mean ± SD)
		Level ^1^	Conc. (g/L) ^2^	Level ^1^	Conc. (g/L) ^2^	
Sweetness	44.6 ± 29.6 ^a,b^	1.6 ± 0.7 ^b,c^	4.78 ± 2.09	3.2 ± 1.2 ^c^	9.81 ± 4.0	4.1 ± 1.9 ^a,b^
Sourness	30.6 ± 23.9 ^c,d^	1.9 ± 0.9 ^a,b^	0.09 ± 0.05	3.7 ± 1.3 ^a,b^	0.19 ± 0.06	4.2 ± 1.6 ^a^
Saltiness	42.7 ± 28.2 ^a,b^	1.5 ± 0.7 ^c^	0.32 ± 0.20	3.4 ± 1.3 ^b,c^	0.97 ± 0.49	2.6 ± 1.5 ^d,e^
Bitterness (caffeine)	23.8 ± 25.9 ^d^	2.3 ± 1.4 ^a^	0.12 ± 0.07	3.9 ± 1.4 ^a^	0.21 ± 0.07	3.5 ± 1.5 ^b,c^
Bitterness (quinine)	36.9 ± 31.8 ^b,c^	2.2 ± 1.3 ^a^	0.002 ± 0.001	3.9 ± 1.3 ^a,b^	0.004 ± 0.002	3.1 ± 1.6 ^d^
Umami	53.8 ± 29.6 ^a^	1.8 ± 1.2 ^b,c^	0.27 ± 0.21	3.7 ± 1.3 ^a,b^	0.99 ± 0.51	2.2 ± 1.4 ^e^

^1^ Mean levels 1–5 (Table 1). ^2^ Mean concentrations in g/L, calculated based on taste compound concentrations corresponding to the mean level (Table 1). ^3^ Mean liking (scale 1–7) was measured on basic taste solutions of Level 5 concentration (Table 1). Different letters in detection and recognition (level) columns indicate a significant difference at *p* < 0.05 from Tukey’s test.

**Table 5:** Fattiness sensitivity measured in milk samples (*n* = 87 subjects).

Pair	Fat Difference	Correctly Identified	Incorrectly Identified	Correctly Identified and Prefer Low-Fat Sample	Incorrectly Identified and Prefer High-Fat Sample
Low	0.5%	49% (*n* = 43)	51% (*n* = 44)	70% (*n* = 30)	68% (*n* = 30)
Medium	1.0%	49% (*n* = 43)	51% (*n* = 44)	60% (*n* = 26)	68% (*n* = 30)
High	1.5%	56% (*n* = 49)	44% (*n* = 38)	61% (*n* = 30)	74% (*n* = 28)

**Table 6:** Overview of key findings across measurement approaches and taste sensations.

Variable	Methods	Tastes	Groups	Gender Effects
Sensitivity	DT positively correlates to RT DT and RT negatively correlate to taste responsiveness (*r* = −0.14/DT, *r* = −0.11/RT, *p* < 0.01) PROP responsiveness positively correlates to taste responsiveness (*r* = 0.13, *p* < 0.01)	Caffeine sensitivity correlates moderately to quinine sensitivity for DT, RT, and responsiveness, respectively (*r* = 0.38, *r* = 0.36, *r* = 0.47, *p* < 0.01) Caffeine and quinine sensitivities (DT, RT, responsiveness) do not correlate with PROP responsiveness Responsiveness to salty and umami, respectively (*r* = 0.26, *r* = 0.24, *p* < 0.05) have positive correlation with PROP responsiveness Responsiveness to quinine is most subject to individual variations Sweetness and bitterness from caffeine easiest tastes to name correctly Saltiness and umami hardest tastes to name correctly	PROP phenotype: 13% nontasters, 51% medium-tasters, 36% supertasters Fat sensitivity: 42.5% fat-sensitive and 52.5% nonsensitive	Boys are marginally less sensitive than girls towards sweetness and bitterness according to DT (*p* = 0.08)
Taste liking	Taste liking does not correlate with taste sensitivity	Fat-sensitive children prefer low-fat milk samples Non-fat-sensitive children prefer high-fat milk samples and state higher liking of fatty foods (*p* = 0.04)	NA	Marginally higher liking score for sweet and bitter tastes (*p* < 0.1) in boys compared to girls
Food liking	Food liking poorly correlates to basic taste sensitivity measures except for Umami sensitivity (DT)	Positively driven by sweetness and fattiness characteristics Negatively driven by bitterness and umami characteristics	Fat-sweet likers (40%), sour likers (28%), bitter-umami likers (32%)	No gender effect observed for food liking

DT = Detection threshold, RT = Recognition threshold, NA = Not applicable.

## Supplementary Materials

The following are available online at https://www.mdpi.com/2304-8158/9/9/1315/s1, Table S1: Food list questionnaire and percentage of children who were familiar to the foods
