# Supplementary material for: Investigating the Relationships between Basic Tastes Sensitivities, Fattiness Sensitivity, and Food Liking in 11-Year-Old Children
Source: Foods. 2020 Sep 18;9(9):1315. doi: 10.3390/foods9091315 (PMC7554820; doi:10.3390/foods9091315)
Supplement: Supplementary file 1 [file foods-09-01315-s001.pdf]

Table 1. Food list and percentage of children who were familiar with the foods.

Food	Taste Profile	Familiarity (Children Who Have Tasted the Food, in %)
Strawberry jam	Sweet	96%
Sweetened soft drink	Sweet	97%
Caramel pudding	Sweet, fatty	70%
Chocolate milk	Sweet, fatty	96%
Sweet bread with filling	Sweet, fatty	91%
Brownies	Sweet, fatty	99%
Doughnut	Sweet, fatty	92%
Ice cream	Sweet, fatty	99%
Brussels sprouts	Bitter	61%
Green onion	Bitter	85%
Spinach	Bitter	66%
Broccoli	Bitter	95%
Dark chocolate	Bitter	97%
Soy sauce	Salty, umami	79%
Cod	Salty, umami	87%
Tomato soup	Salty, umami, fatty	94%
Fish fingers	Salty, umami, fatty	93%
Fish cake	Salty, umami	92%
Sausage	Salty, fatty	97%
Hamburger	Salty, fatty	98%
Pizza	Salty, fatty	96%
Chips	Salty, fatty	98%
Soft cheese	Salty, fatty	91%
Butter	Fatty	94%
Avocado	Fatty	78%
Blueberry	Sour	96%
Kiwi	Sour	87%
Mandarin	Sour	92%
Rhubarb	Sour	67%
Sour candy	Sour	97%
**Taste**	**N**	**Average Familiarity** (% of children) *
Sweet	8	92.5%
Sour	5	90.0%
Salty	10	92.2%
Bitter	5	81.1%
Umami	5	88.0%
Fatty	15	92.3%

* Average percentage of children who have tasted the N foods from the food list representing sweet, sour, salty, bitter, umami, and fatty taste, respectively.
